# Is the involvement of opinion leaders in the implementation of research findings a feasible strategy?

**DOI:** 10.1186/1748-5908-1-3

**Published:** 2006-02-22

**Authors:** Jeremy M Grimshaw, Martin P Eccles, Jenny Greener, Graeme Maclennan, Tracy Ibbotson, James P Kahan, Frank Sullivan

**Affiliations:** 1 Health Services Research Unit, University of Aberdeen, Aberdeen, UK; 2 Centre for Health Services Research, University of Newcastle upon Tyne, Newcastle, UK; 3 RAND EUROPE, Leiden, Netherlands; 4 NHS Tayside Professor of Research & Development in General Practice and Primary Care, Community Health Sciences Division, University ofDundee, Dundee, UK

## Abstract

**Background:**

There is only limited empirical evidence about the effectiveness of opinion leaders as health care change agents.

**Aim:**

To test the feasibility of identifying, and the characteristics of, opinion leaders using a sociometric instrument and a self-designating instrument in different professional groups within the UK National Health Service.

**Design:**

Postal questionnaire survey.

**Setting and participants:**

All general practitioners, practice nurses and practice managers in two regions of Scotland. All physicians and surgeons (junior hospital doctors and consultants) and medical and surgical nursing staff in two district general hospitals and one teaching hospital in Scotland, as well as all Scottish obstetric and gynaecology, and oncology consultants.

**Results:**

Using the sociometric instrument, the extent of social networks and potential coverage of the study population in primary and secondary care was highly idiosyncratic. In contrast, relatively complex networks with good coverage rates were observed in both national specialty groups. Identified opinion leaders were more likely to have the expected characteristics of opinion leaders identified from diffusion and social influence theories. Moreover, opinion leaders appeared to be condition-specific. The self-designating instrument identified more opinion leaders, but it was not possible to estimate the extent and structure of social networks or likely coverage by opinion leaders. There was poor agreement in the responses to the sociometric and self-designating instruments.

**Conclusion:**

The feasibility of identifying opinion leaders using an off-the-shelf sociometric instrument is variable across different professional groups and settings within the NHS. Whilst it is possible to identify opinion leaders using a self-designating instrument, the effectiveness of such opinion leaders has not been rigorously tested in health care settings. Opinion leaders appear to be monomorphic (different leaders for different issues). Recruitment of opinion leaders is unlikely to be an effective general strategy across all settings and professional groups; the more specialised the group, the more opinion leaders may be a useful strategy.

## Background

Despite the considerable resources devoted to biomedical science, a consistent finding from the literature is that the transfer of research findings into practice is a slow and haphazard process. For many years, the traditional approach to dissemination has been the publication of research findings in journals (or other media), which the target audience is likely to read, in the belief that this will lead to changes in practice. The recognition of the failure of this model has led to greater awareness of the role of other factors in the practice environment influencing behaviour [1] and the importance of identifying potential barriers to changing practice when planning implementation activities [2].

Mittman and colleagues [3] noted that health care professionals work within peer groups, which share common beliefs and assumptions and group norms, and that individual behaviour can be strongly influenced by these factors. They identified a number of strategies to facilitate the implementation of research findings by using these social influences. One strategy generating considerable interest is the use of opinion leaders.

Opinion leadership (more properly termed Informal Opinion Leadership; for ease of reading we refer to 'opinion leadership' throughout this article) is the degree to which an individual is able to influence other individuals' attitudes or overt behaviour informally, in a desired way with relative frequency [4]. This informal leadership is not a function of the individual's formal position or status in the system; it is earned and maintained by the individual's technical competence, social accessibility, and conformity to the system's norms. When compared to their peers, opinion leaders tend to be more exposed to all forms of external communication, have somewhat higher social status, and to be more innovative. However, the most striking feature of opinion leaders is their unique and influential position in their system's communication structure; they are at the centre of interpersonal communication networks – interconnected individuals who are linked by patterned flows of information.

There is only limited empirical evidence about the effectiveness of opinion leaders as health care change agents. Thomson and colleagues [5] identified only eight rigorous evaluations of opinion leaders in the health care literature. Six out of seven trials observed improvements in at least one process of care variable, although these results were only statistically and clinically important in two trials. One of three trials measuring patient outcomes observed an improvement that was of practical importance. They concluded that using local opinion leaders resulted in mixed effects and that further research was required before the widespread use of this intervention could be justified.

There are four approaches to the measurement of opinion leadership: sociometric methods, key informant methods, self-designating methods, and observation [4]. Sociometric methods [4,6] involve extensive analyses of leadership nominations within members of a peer group. Seven out of the eight opinion leader trials used a sociometric instrument developed by Hiss, [6] which seeks nominations for individuals who are knowledgeable, good communicators and have humanistic philosophies. Key informant methods ask a small(er) number of individuals, who are particularly knowledgeable about a network, to identify individuals who serve as main sources of information, influence or both. This method was used by the other trial. Self-designating methods [7] involve self-reporting, by all members of a network, of their own role as an opinion leader. This method has been used to identify individuals for marketing exercises and for studies promoting individual behaviour change; however, it has not be used to identify opinion leaders in health care professional groups. Observation methods involve direct observation and work best in small systems.

Although using opinion leaders to induce the rank-and-file to change behaviour has great intuitive appeal, we believe that a number of conditions are prerequisite to its use as an effective strategy. Firstly, there must be effective interpersonal communication networks. Secondly peer influence must work amongst professional groups. Thirdly, opinion leaders must be readily identifiable. And finally, the leaders must be inclined to adopt changes based on evidence, so that they can honestly influence others. Support for these four prerequisites is encouraging but not definitive. In some professional groups, it may be difficult to identify opinion leaders, or the group may be so diffuse that there are few opportunities for influence (un-cohesive or ineffective interpersonal networks). A further complicating factor is the uncertainty about whether – in any professional social network – there will be one set of all-purpose opinion leaders (polymorphism) or whether there are different opinion leaders for different issues (monomorphism).

The current study aimed to: examine the feasibility of identifying opinion leaders in different professional groups within the United Kingdom (UK) National Health Service using two different instruments, a sociometric instrument [6] and a self-designating instrument [7]; to describe the professional and personal characteristics of the opinion leaders so identified; and to determine whether opinion leaders are inclined to adopt changes based on evidence.

## Methods

The study involved postal surveys of different professional groups in different geographical areas in Scotland.

### Study sites and populations

Study sites were chosen for administrative ease. In primary care, we surveyed all general practitioners (Primary Care Doctors), practice nurses (nurses working in and employed by general practices), and practice managers in two regions of Scotland, one Health Board in the West of Scotland (PC1), and one in the North East of Scotland (PC2). In secondary care, we surveyed all medical and surgical junior hospital doctors (secondary care doctors in training grades), consultants (hospital specialists), and nursing staff in two district general hospitals and one teaching hospital in Scotland. One of the district general hospital sites was in the West of Scotland (DGH1); the other district general hospital (DGH2) and the teaching hospital (TH) were both in the North East of Scotland. Finally, we surveyed two national specialty groups – all Scottish Obstetric and Gynaecology consultants, and all Scottish Oncology consultants. All permissions and contact details were obtained from the relevant administrative bodies.

### Survey instrument

Full details of the instruments are reported elsewhere [8]. In summary the questionnaire consisted of four sections:

*1. Personal and professional characteristics*,

*2. Ways of keeping up to date with findings from research*,

*3. Types of clinical effectiveness information used *(Questions adapted from material developed by Elisabeth West and colleagues, personal communication), and

4. Identification of opinion leaders via two methods:

*a*) *Sociometric instrument *– adapted from the Hiss [6] instrument, there were three questions each seeking up to three nominations for individuals who were knowledgeable, good communicators and humanistic (see Table 1).

**Table 1:** Generic sociometric instrument used in surveys

We are trying to identify colleagues who, by virtue of their views, knowledge or standing, are used as a source of advice by their peers.	
Please read each of the paragraphs and write in the names of up to three colleagues that best fit the description of each characteristic. The same person may be named for more than one characteristic. You can name anyone with whom you come into regular contact.	
1.	These colleagues express themselves clearly and concisely, giving practical information. They take the time to answer you completely, and do not leave you with the feeling that they were too busy to answer your inquiry.
2.	These colleagues are up-to-date and demonstrate a command of knowledge about clinical issues in general practice.
3.	These colleagues are caring and demonstrate a high level of concern. They never talk down to you; they treat you as an equal.

*b*) *Self-designating instrument *– adapted from the Childers [7] instrument, there were six questions which respondents had to rate on a 1 – 5 scale (Table 2). The direction of response was reversed for questions 2, 4, and 6.

**Table 2:** Generic self-designating questionnaire used in surveys.

This section is about the degree to which you advise colleagues with whom you come into contact. Please rate yourself on the following scales relating to your interactions with colleagues regarding clinical issues in general practice, by circling the number which you feel is most appropriate.				
**1. In general, do you talk to your colleagues about issues in general practice?**				
Very often				Never
5	4	3	2	1
**When you talk to your colleagues about clinical issues in general practice, do you:**				
Give very little information				Give a lot of information
5	4	3	2	1
**In the past six months, how many times have you given information to colleagues about clinical issues in general practice?**				
Many times				Never
5	4	3	2	1
**Compared with your colleagues, how likely are you to be asked about clinical issues in general practice?**				
Not at all likely to be asked				Very likely to be asked
5	4	3	2	1
**In a discussion of clinical issues in general practice, which of the following happens most often?**				
You tell your colleagues about your ideas				Your colleagues tell you about their ideas
5	4	3	2	1
**Overall in your discussions with colleagues about clinical issues in general practice, are you:**				
Not used as a source of advice				Often used as a source of advice
5	4	3	2	1

**Table 3:** Conditions chosen for condition-specific instruments

Target group	Condition
**Primary care**	
General practitioners	Ischaemic heart disease
Practice nurses	Ischaemic heart disease
Practice managers	N/A
**Secondary care**	
Physicians	Ischaemic heart disease
Surgeons	Laparoscopic surgery
Medical nursing staff	Management of pressure sores
Surgical nursing staff	Post operative pain relief
**National specialty groups**	
Obstetrics and gynaecology	Laparoscopic surgery
Oncology	Management of breast cancer

We asked each target group to complete questionnaires to identify both generic and condition-specific opinion leaders with the exception of practice managers, who were not asked to identify condition-specific opinion leaders, as these were exclusively clinical. For example, we asked the national sample of obstetricians and gynaecologists to identify opinion leaders for general gynaecological issues and opinion leaders for issues about the use of Laparoscopic surgical techniques. The conditions chosen for each target group are given in Box 3.

### Survey procedure

Study subjects were sent an initial questionnaire and cover letter explaining the study. Non-responders were sent a reminder at two weeks. Respondents returning blank questionnaires were not sent reminders and were treated as non-respondents.

### Analysis

Data were analysed using SPSS or Arcus Biostat. For the purposes of the analysis of the sociometric instrument, an individual nominated in all three questions by at least two respondents was classified as a 'sociometric opinion leader' (SOL). We calculated the aggregated 'instrument respondent coverage' of the identified SOLs (the percentage of respondents completing the sociometric instrument who reported being influenced by the identified SOLs) and the maximum coverage of any individual SOL. This is likely to be the best-case scenario, as it assumes that similar proportions of non-respondents would be covered by SOLs; whereas, it is likely that non-responders or responders who did not complete the sociometric instrument were less likely to be influenced by SOLs. As a sensitivity analysis, we also calculated the 'study population coverage' (the percentage of the total sample influenced by the identified SOLs). This represents a worse case scenario and assumes that the respondents who did not complete the sociometric questionnaire and non-respondents were not able to identify SOLs.

The total score across the self-designating instrument questions was summed. Respondents scoring within the top 20% were classified as 'self designated opinion leaders' (SDOLs) to allow a reasonable split for statistical analysis. It was not possible to identify the potential coverage of these identified opinion leaders, and potential opinion leaders external to the sample could not be identified.

#### Characteristics of opinion leaders

We tested the convergent validity of the identifying instruments by testing whether identified individuals were more likely than other respondents to possess expected characteristics of opinion leaders (identified from diffusions and social influence theories). The following hypotheses were tested: Social network related – Opinion Leaders were more likely to have trained locally (and thus have more developed local social networks), and were more likely to belong to professional groups; Experience related – Opinion Leaders were more likely to have been qualified for longer, and were more likely to be in senior posts; Keeping up-to-date – Opinion Leaders were more likely to have professional and academic qualifications, to have higher keeping up-to-date scores, and be more likely to use effectiveness materials.

The number of SOLs identified in any individual survey was small. Therefore, to maximise statistical power, we combined datasets across survey samples wherever possible. [All datasets did not contribute to all analyses as the specific questions relating to personal and professional characteristics varied across professional groups.] Chi square tests (for categorical data) and T-tests (for continuous data) were undertaken to test these hypotheses. The results for categorical data are expressed as odds ratios with 95% confidence intervals and associated significance tests.

#### Other analyses

We undertook analyses to examine whether in any professional social network there was one set of all-purpose opinion leaders (polymorphism), or whether there were different opinion leaders for different issues (monomorphism). We examined the likelihood that generic SOLs were also identified as condition-specific SOLs, within the same professional network, by treating the two instruments as if they were diagnostic tests. We calculated the inter-test agreement and the sensitivity, and the specificity and positive predictive value of the generic instrument compared to the condition-specific instrument (treated as the 'gold standard').

We also compared the potential coverage of generic SOLs identified as condition-specific SOLs to the potential coverage of all the condition-specific SOLs within the same network. Similarly, we examined the likelihood that generic SDOLs also identified themselves as condition-specific SDOLs within the same network. However, due to the method of identification we were unable to compare the likely coverage of generic SDOLs identified as condition-specific SDOLs with all the condition-specific SOLs within the same network.

#### Comparison of different identification methods

Similarly, we examined the likelihood that generic SOLs were also generic SDOLs and that condition-specific SOLs were also generic SDOLs. We again calculated the inter-test agreement and the sensitivity, specificity and positive predictive value of the self-designating instrument compared to the sociometric instrument (treated as the 'gold standard').

## Results

### Survey response rates

Overall survey response rates are shown in Table 4. Primary care response rates were lower from general practitioners compared to practice nurses [55.7% (316/567) vs. 70.1% (188/268) respectively, Chi square 15.81, df = 1, p < 0.0001]. Secondary Care response rates varied across sites [DGH1 42.5% (82/193), DGH2 58.2% (70/120) and TH 48.2% (145/301), Chi square 7.45 df = 2, p < 0.05]. Response rates from secondary care surveys were lower compared to primary care [48.4% (297/614) vs. 60.8% (594/977), Chi square 26.27, df = 1, p < 0.0001], although secondary care survey respondents were more likely than primary care survey respondents to complete the sociometric instruments [68.0% (202/297) vs. 57.2% (340/594), Chi square 9.65, d f= 1, p < 0.01]. For the national specialty groups, the overall response rate was 73.3% (143/195). This response rate was higher than those for both primary care [60.8% (594/977) Chi square 10.94, df = 1, p < 0.001] and secondary care [48.4% (297/614) Chi square 37.17, df = 1, p < 0.0001]. Respondents from national specialty groups also were more likely to complete the generic sociometric instruments than the primary care survey [74.8% (107/143) vs. 57.2% (340/594) primary care survey respondents, Chi square 14.93, df = 1, p < 0.001]. Respondents from national specialty groups also were more likely to complete the condition-specific sociometric instruments than the primary care and secondary care survey respondents [76.2% (109/143) vs. 41.9% (249/504) primary care, Chi square 32.66, df = 1, p < 0.0001; 76.2% (109/143) vs. 57.2% (170/297) secondary care, Chi square 14.99, df = 1, p < 0.0001].

**Table 4:** Response rates

	Total mailed	Total returned (% total mailed)	Attempted generic sociometric instrument (% respondents)	Attempted condition-specific sociometric instrument (% respondents)
PC1				
General practitioners	211	86 (40.6%)	40 (46.5%)	37 (43.0%)
Practice nurses	66	37 (56.1%)	16 (43.2%)	16 (43.2%)
Practice managers	62	32 (51.6%)	21 (65.6%)	N/A
Total	339	155 (45.7%)	77 (49.7%)	53 (43.1%)
PC2				
General practitioners	356	230 (64.6%)	130 (56.5%)	111 (48.3%)
Practice nurses	202	151 (74.6%)	98 (64.9%)	85 (56.3%)
Practice managers	80	58 (72.5%)	35 (60.3%)	N/A
Total	638	439 (68.8%)	263 (59.9%)	196 (51.4%)
DH1				
Surgeons	41	21 (51.2%)	14 (66.7%)	8 (38.1%)
Physicians	33	22 (66.7%)	19 (86.4%)	16 (72.7%)
Surgical nurses	41	9 (22.0%)	6 (66.7%)	6 (66.1%)
Medical nurses	78	30 (38.5%)	21 (70.0%)	18 (60.0%)
Total	193	82 (42.5%)	60 (73.2%)	48 (58.5%)
DH2				
Surgeons	11	7 (63.6%)	6 (85.7%)	3 (42.9%)
Physicians	10	4 (40.0%)	4 (100.0%)	4 (100.0%)
Surgical nurses	53	34 (64.2%)	32 (94.1%)	28 (82.4%)
Medical nurses	46	25 (54.3%)	13 (52.0%)	14 (56.0%)
Total	120	70 (58.2%)	55 (78.6%)	49 (70.0%)
TH				
Surgeons	35	18 (51.4%)	11 (61.1%)	8 (44.4%)
Physicians	119	51 (42.9%)	31 (60.8%)	23 (45.1%)
Surgical nurses	89	37 (41.6%)	13 (35.1%)	14 (40.0%)
Medical nurses	58	39 (67.2%)	32 (82.1%)	28 (71.8%)
Total	301	145 (48.2%)	87 (60.0%)	73 (50.3%)
National specialty groups				
Obstetricians and gynaecologists	151	108 (71.5%)	78 (72.2%)	81 (75.0%)
Oncologists	45	35 (77.7%)	29 (82.6%)	28 (80.0%)
Total	195	143 (73.3%)	107 (74.8%)	109 (76.2%)

### Identification of opinion leaders

The response for the sociometric instrument from primary care, secondary care, and national networks are shown in Tables 5 and 6. Tables 7 and 8 summarise the mean instrument scores for all respondents, and generic and condition-specific self-designating opinion leaders.

**Table 5:** Summary of primary care responses to sociometric instrument

Survey sample	Number of SOLs identified	Instrument respondent coverage	Maximum individual SOL coverage	Population respondent coverage	Comments
Generic					
General practitioners					
PC1	1	5.0%	5.0%	1.0%	Single, within practice nominations
PC2	10	14.6%	2.3%	5.3%	Mainly, within practice nominations
Practice nurses					
PC1	1	18.8%	18.8%	4.6%	Single, within practice nomination
PC2	17	28.6%	4.1%	13.9%	Mainly, within practice nominations
Practice managers					
PC1	2	19.1%	9.5%	6.5%	Limited across practice network
PC2	4	25.7%	11.4%	11.3%	Limited across practice network
Condition-specific					
General practitioners					
PC1	4	40.5%	32.4%	7.1%	Relatively simple network, with modest coverage from cardiologists
PC2	9	27.9%	15.3%	11.9%	Relatively simple network, with modest coverage from cardiologists
Practice nurses					
PC1	0	0%	0%	0%	No SOL identified
PC2	14	28.2%	2.4%	8.7%	Mainly, within practice nominations

**Table 6:** Summary of secondary care and national network responses to sociometric instrument

Survey sample	Number of SOLs identified	Instrument respondent coverage	Maximum individual SOL coverage	Population respondent coverage	Comments
Generic Surgeons					
DGH1	1	50%	50%	17.1%	Single SOL identified
DGH2	0	0%	0%	0%	No SOLs identified
TH	1	27.2%	27.2%	8.6%	Single SOL identified
Physicians					
DGH1	3	26.3%	21.1%	15.2%	Simple network
DGH2	0	0%	0%	0%	No SOLs identified
TH	2	12.9%	6.5%	3.4%	Simple network
Nurses					
DGH1	2	14.8%	7.4%	3.4%	Simple network, within ward nominations
DGH2	11	57.8%	15.6%	26.3%	Simple network, mainly within ward nominations
TH	6	33.3%	33.3%	10.2%	Simple network, within ward nominations
Condition-specific Surgeons					
DGH1	1	87.5%	87.5%	17.1%	Single SOL identified
DGH2	0	0%	0%	0%	No SOL identified
TH	2	50%	37.5%	11.4%	Simple network
Physicians					
DGH1	1	12.5%	12.5%	6.1%	Single SOL identified
DGH2	0	0%	0%	0%	No SOL identified
TH	7	47.8%	21.7%	9.2%	Simple network
Surgical nurses					
DGH1	1	33.3%	33.3%	4.9%	Single SOL identified
DGH2	10	62.5%	25.0%	27.7%	Complex network, mainly within ward nominations
TH	6	85.7%	35.7%	13.5%	Complex network, within ward nominations and across ward nominations for specialist nurse teams
Medical nurses					
DGH1	1	11.1%	11.1%	2.6%	Single SOL identified
DGH2	2	50.0%	42.9%	15.2%	Simple network
TH	4	46.4%	28.6%	22.4%	Simple network, within and across ward nominations for specialist nurse teams
Generic					
Obstetrics and gynaecology	20	46.2%	7.7%	23.8%	Complex network within and across centres
Oncology	4	34.5%	13.8%	22.2%	Limited across centre network
Condition-specific					
Obstetrics and gynaecology	14	48.2%	17.3%	25.9%	Complex within and across centre network
Oncology	9	53.6%	17.9%	33.3%	Mainly within centre networks

**Table 7:** Summary of generic self-designating instrument responses

Survey sample	Total respondents	Mean score of all respondents (SD)	Range of scores of all respondents (SD)	Total SDOLs	Mean score of self-designating opinion leaders (SD)	Range of scores of self-designating opinion leaders (SD)
General practitioners						
PC1	78	19.96 (4.03)	9–30	16	25.31 (1.85)	23–30
PC2	222	20.36 (3.74)	10–30	47	25.55 (1.47)	24–30
Practice nurses						
PC1	35	21.60 (4.69)	13–30	7	28.43 (0.79)	28–30
PC2	144	21.01 (4.04)	4–30	29	26.34 (1.72)	22–30
Practice managers						
PC1	32	20.50 (4.68)	10–29	7	26.71 (1.50)	25–29
PC2	56	16.80 (2.57)	10–22	13	19.69 (1.03)	19–22
Surgeons						
DGH1	16	20.13 (3.69)	13–25	6	23.67 (0.82)	23–25
DGH2	7	22.57 (5.16)	16–29	1	29.00 (0.00)	29–29
TH	18	21.33 (5.39)	11–30	5	27.20 (1.79)	26–30
Physicians						
DGH1	21	19.38 (5.53)	6–27	4	23.75 (3.20)	21–27
DGH2	3	23.33 (3.51)	20–27	1	27.00 (0.00)	27–27
TH	47	21.15 (4.62)	2–27	12	25.42 (1.88)	20–27
Surgical nurses						
DGH1	9	20.89 (4.11)	16–29	4	20.00 (2.31)	18–22
DGH2	34	21.32 (3.87)	12–29	11	25.73 (1.85)	24–29
TH	37	19.62 (4.02)	5–27	7	24.86 (1.35)	23–27
Medical nurses						
DGH1	30	19.90 (4.84)	6–27	7	25.14 (1.07)	24–27
DGH2	25	21.04 (3.60)	15–28	7	25.57 (1.51)	24–28
TH	34	21.50 (3.17)	15–28	9	25.44 (1.74)	23–28
Obstetricians and Gynaecologists	102	23.08 (3.71)	10–30	20	28.0 (1.08)	27–30
Oncologists	33	24.42 (3.87)	13–29	10	28.40 (0.52)	28–29

**Table 8:** Summary of condition-specific, self-designating instrument responses

Survey sample	Total respondents	Mean score of all respondents (SD)	Range of scores of all respondents (SD)	Total SDOLs	Mean score of self-designating opinion leaders (SD)	Range of scores of self-designating opinion leaders (SD)
General practitioners						
PC1	77	16.69 (4.19)	4–30	15	22.80 (2.96)	20–30
PC2	216	17.69 (4.34)	1–30	36	23.86 (2.22)	22–30
Practice nurses						
PC1	32	16.91 (5.87)	5–28	7	24.14 (2.12)	22–28
PC2	139	16.48 (5.27)	1–30	27	23.33 (2.27)	21–30
Surgeons						
DGH1	12	16.50 (7.17)	5–27	5	23.40 (3.21)	20–27
DGH2	7	16.29 (8.42)	5–26	2	26.00 (0.00)	26–26
TH	16	16.69 (7.85)	6–30	3	28.33 (1.53)	27–30
Physicians						
DGH1	21	17.81 (5.26)	7–26	6	23.50 (1.76)	22–26
DGH2	3	22.00 (4.00)	18–26	1	26.00 (0.00)	26–26
TH	45	16.87 (6.11)	6–30	9	25.8 (2.98)	21–30
Surgical nurses						
DGH1	9	21.33 (2.65)	18–27	3	20.67 (2.31)	18–22
DGH2	34	21.50 (3.73)	11–28	7	26.71 (0.76)	26–28
TH	35	20.23 (4.31)	7–29	11	24.82 (2.14)	23–29
Medical nurses						
DGH1	29	20.97 (4.56)	12–28	7	26.71 (1.25)	25–28
DGH2	25	19.68 (4.22)	9–28	5	25.40 (2.70)	21–28
TH	37	18.81 (4.57)	7–27	7	25.71 (1.11)	24–27
Obstetricians and Gynaecologists	100	16.45 (6.04)	5–30	18	25.28 (2.11)	23–30
Oncologists	31	21.16 (5.54)	12–29	6	28.00 (0.89)	27–29

### Characteristics of opinion leaders

We tested whether identified generic and condition-specific SOLs and SDOLs were more likely to have expected characteristics of opinion leaders than other respondents. The results are summarised in Table 9. Generic SOLs were more likely to: belong to professional groups, have been qualified longer, be in a senior position, and have high effectiveness and keeping-up-to-date scores. Condition-specific SOLs were more likely to belong to professional groups and be in a senior position; they were less likely to have attended a local medical school. Generic SDOLs were more likely to belong to professional groups, be in a senior post, have more qualifications, and high effectiveness and keeping-up-to-date scores. Condition-specific SDOLs were more likely to have high effectiveness and keeping-up-to-date scores. Thus, all classes of opinion leaders had some of the expected characteristics of opinion leaders. However, the odds ratio and difference in mean up-to-date scores were generally higher in generic and condition-specific SOLs compared with SDOLs.

**Table 9:** Characteristics of identified opinion leaders (odds ratios with 95% confidence intervals)

Hypothesis	Generic sociometric	Condition-specific sociometric	Generic self-designating	Condition-specific self-designating
Social network related				
OLs more likely to belong to professional groups	5.27 (2.38 – 11.65)****	3.90 (1.63 – 9.33)**	1.56 (1.13 – 2.17)**	1.13 (0.79 – 1.58)
OLs more likely to have attended local medical school	1.32 (0.62 – 2.82)	0.41(0.08 – 0.90)***	1.02 (0.65 – 1.54)	0.87 (0.55 – 1.38)
Experience related				
OLs more likely to have been qualified longer	1.90 (1.10 – 3.28)**	1.18 (0.64 – 2.20)	0.99 (0.72 – 1.36)	1.20 (0.85 – 1.69)
OLs more likely to be in senior posts	6.69 (2.33 – 19.20) ***	5.72 (1.69 – 19.34)***	2.02 (1.23 – 3.21)***	1.35 (0.85 – 2.15)
Qualifications				
OLs more likely to have qualifications	1.05 (0.6 3 – 1.75)	1.27 (0.68 – 2.36)	1.80 (1.33 – 2.44)***	0.96 (0.68 – 1.36)
Other				
OLs more likely to spend time teaching	0.88 (0.16 – 4.74)	1.35 (0.31 – 5.98)	.93 (0.79 – 4.67)	0.92 (0.34 – 2.50)
OLs more likely to spend time on research	2.30 (0.49 – 10.92)	1.82 (0.41 – 8.11)	2.14 (0.86 – 5.34)	1.10 (0.40 – 3.04)
Keeping up to date score				
Mean Opinion Leader Score	3.57	3.47	3.48	3.40
Mean score of other respondents	3.29	3.30	3.25	3.27
Mean difference in up-to-date score	0.28	0.17	0.23	0.13
95% CI and significance^+^	(0.14 – 0.43)**	(-0.09 – 0.36)	(0.14 – 0.32)***	(0.03 – 0.24)*
Use of clinical effectiveness materials score				
Mean Opinion Leader Score	2.58	2.37	2.53	2.58
Mean score of other respondents	2.38	2.42	2.36	2.38
Mean difference in up-to-date score	0.3	-0.05	0.17	0.20
95% CI and significance^+^	(-0.02 – 0.41)	(-0.33 – 0.21)	(0.04 – 0.30)*	(0.04 – 0.30)*

Key – * – p < 0.05, ** – p < 0.01, *** – p < 0.001, **** – p < 0.0001, + Independent samples t-test

### Monomorphism versus polymorphism

#### Sociometric instruments

Across all surveys, 81 generic SOLs and 86 condition-specific SOLs were identified; 19 individuals were identified as both generic and condition-specific SOLs (Table 10). The inter-instrument agreement was only fair (unweighted kappa = 0.20). The sensitivity and specificity of the generic instrument to identify condition-specific SOLs was 27.4% and 93.0%, respectively. The positive predictive value of the generic instrument for identifying condition-specific SOLs was 26.4%. Condition-specific SOL coverage rates were greater than generic SOLs coverage rates in the majority of surveys (Tables 5 and 6).

**Table 10:** Agreement between sociometric and self-nominating instruments for generic and condition-specific opinion leadership

Sociometric Instrument Generic vs. condition-specific Opinion Leadership			
	Condition-specific instrument		
		
	Opinion leader	Not opinion leader	
Generic instrument			
Opinion leader	23	64	87
Not opinion leader	61	856	917
	84	920	1001
Self-designating Instrument Generic vs. condition-specific Opinion Leadership^1^			
	Condition-specific instrument		
		
	Opinion leader	Not opinion leader	

Generic instrument			
Opinion leader	77	116	193
Not opinion leader	93	563	656
	170	679	849
Generic Opinion Leadership sociometric vs. self-designating instrument^1^			
	Self-designating instrument		
		
	Opinion leader	Not opinion leader	

*Sociometric instrument*			
Opinion leader	23	37	60
Not opinion leader	200	720	920
	223	757	980
Condition-specific Opinion Leadership sociometric vs. self-designating instrument^1^			
	Self-designating instrument		
		
	Opinion leader	Not opinion leader	

*Sociometric instrument*			
Opinion leader	26	15	41
Not opinion leader	149	678	827
	175	693	868

1. Analysis limited to respondents with both generic and condition-specific instruments completed.

#### Self-designating instruments

Across all surveys, 193 generic SDOLs and 170 condition-specific SDOLs were identified; 77 individuals were identified as both generic and condition-specific SDOLs (Table 10). The inter-instrument agreement was only fair (unweighted kappa = 0.27). The sensitivity and specificity of the generic instrument to identify condition-specific SDOLs were 45.3% and 82.9% respectively. The positive predictive value of the generic instrument for identifying condition-specific SDOLs was 39.9%. It was not possible to calculate the coverage rate of SDOLs.

### Comparison of identification methods

#### Generic instruments

Across all surveys a maximum of 87 generic SOLs and 223 generic SDOLS were identified, 23 individuals were identified as both generic SOLs and SDOLs (Table 10). The inter-instrument agreement was poor (unweighted kappa = 0.07). The sensitivity and specificity of the generic self-designating instrument to identify generic SOLs was 38.3% and 78.3% respectively. The positive predictive value of the generic instrument for identifying condition-specific SDOLs was 10.3%. Furthermore, the condition-specific coverage rates of the generic SOLs were substantially lower than the condition-specific coverage rates of condition-specific SOLs in all but two surveys, both of which had only identified a single opinion leader (Table 11).

**Table 11:** Condition-specific coverage rates of generic sociometric opinion leaders

Survey sample	Professional group	Condition-specific coverage rates by generic SOLs	Condition-specific coverage of all idenfitied SOLs
PC1	General practitioners	0.0%	40.5%
	Practice nurses	0.0%	0.0%
PC2	General practitioners	2.7%	27.9%
	Practice nurses	12.9%	28.2%
DGH1	Surgeons	87.5%	87.5%
	Physicians	0.0%	12.5%
	Surgical nurses	0.0%	33.3%
	Medical nurses	0.0%	11.1%
DGH2	Surgeons	0.0%	0.0%
	Physicians	0.0%	0.0%
	Surgical nurses	46.4%	62.5%
	Medical nurses	50.0%	50.0%
TH	Surgeons	37.5%	50.0%
	Physicians	8.7%	47.8%
	Surgical nurses	21.4%	85.7%
	Medical nurses	10.7%	46.4%
Obstetrics and gynaecology		21.0%	48.1%
Oncology		0.0%	53.6%

#### Self-designating instruments

Across all surveys, 84 condition-specific SOLs and 175 condition-specific SDOLS were identified, 26 individuals were identified as condition-specific SOLs and SDOLs (Table 11). The inter-instrument agreement was poor (unweighted kappa = 0.18). The sensitivity and specificity of the condition-specific, self-designating instrument to identify condition-specific SOLs was 63.4% and 82.0%, respectively. The positive predictive value of the generic instrument for identifying condition-specific SDOLs was 14.8%.

## Discussion

In this study, we have used two different 'off-the-shelf' methods of identifying opinion leaders across a range of different professional groups in the UK. The study utilised existing instruments that had previously been validated in cross sectional surveys and in randomised trials. The study used replicated surveys across different types of professionals within the UK, which allowed us to identify wide variations across different professional groups and sites in the extent of nominating SOLs and the complexity of networks. Furthermore, this has been one of the first studies to examine whether opinion leaders are polymorphic or monomorphic.

Responses to the sociometric instruments demonstrated a wide variation across different professional groups and sites in the extent of nominating SOLs and the complexity of social networks [8]. These results suggest that the extent of social networks and potential coverage of the study population in primary and secondary care is highly idiosyncratic, and adequate coverage rates cannot be assumed. In contrast, relatively complex networks with good coverage rates were observed in both national specialty groups.

Both SOLs and SDOLs had characteristics of opinion leaders although the odds ratios and mean differences in continuous variables were higher in SOLs. Approximately one-third of generic SOLs also were nominated as condition-specific SOLs, and the condition-specific coverage rate of these SOLs was poor. Similarly, generic SDOLs were relatively unlikely to identify themselves as condition-specific SDOLs. These results suggest that opinion leaders are monomorphic, and that separate identification exercises would be needed for different conditions.

Case studies frequently identify the importance of individuals (opinion leaders, change agents, product champions) in leading and supporting change in the health service. However, these terms are not necessarily well defined, nor mutually exclusive. In this study there was poor agreement in the responses to the sociometric and self-designating instruments. SDOLs were relatively unlikely to have been identified as SOLs and vice versa. There are at least two possible interpretations of this. If the instruments are trying to identify the same construct of opinion leaders, one is performing poorly. Alternatively, the instruments may be identifying different constructs of opinion leaders. The sociometric instrument was rigorously developed [6] and has face validity, but remains the only instrument of its type and thus has not been validated against a comparable instrument. It emphasises opinion leaders who are knowledgeable, humanistic, and good communicators – characteristics identified by physicians as likely to influence their choice of educational influential (Table 1). Work in Norway [9] showed that general practitioners supported the concepts espoused in the sociometric instrument. The instrument demonstrates the extent of social networks and coverage of identified opinion leaders and has been successfully used to identify opinion leaders in randomised trials, which have demonstrated behaviour change. The self-designating instrument emphasises opinion leaders who are commonly consulted by colleagues and who give a lot of information (Table 2), and while the sociometric instrument may identify one construct of opinion leader, other types of leadership also may be influential (e.g., professional or academic leaders). However, there is scope for further exploration of the validity of the self-designating instrument within professional settings. These considerations highlight the potential conceptual and terminological confusion surrounding opinion leadership. Whilst this term is used in a specific technical way within the diffusions of innovation, marketing and social influence literatures, it is commonly used to describe any influential individual (educational, academic or political).

Response rates to the survey overall were moderate (57.8%). The response rate to the sociometric instrument was lower. During pilot work for this study, interviews with primary care respondents – after they had completed the instruments – suggested that they had some difficulties with the concept of opinion leaders, and the questionnaire was also seen as being rather abstract [8]. We have identified eleven studies that have used the sociometric instrument from the systematic review by Thomson, [5] and a forward citation search for the original study by Hiss and colleagues (1978). The majority of previous studies provided inadequate details of the methods of identifying opinion leaders, partly due to editorial pressures on space (Soumerai S, personal communication.). The number of opinion leaders identified varied. In the studies by Stross [10-12]], Lomas [13] and Soumerai [14], the individual with the greatest number of nominations per institution was identified as an opinion leader. In the other studies, a larger number of opinion leaders were identified (similar to the current study). These differences are probably due to different strategies for analysing the sociometric instrument. Coverage rates are rarely reported, although Lomas [13] and Soumerai [14] both report that the identified opinion leaders received the clear majority of votes within their hospital. As a result, it is difficult to assess the coverage likely to be needed if the strategy is successful. All of these factors have important implications for the utility of the method in a service setting, as it would be difficult to justify as a single strategy a method that potentially only drew on just more than half of the population and could not cover the non-responding half. We used convenience samples for this work, so it is important that the study is replicated in other settings and populations of clinicians. Indeed, it would be interesting to repeat it in the same populations in a few years to see if recent UK health reforms, with their emphasis on localities of general practitioners, have changed the situation.

The concept of opinion leadership has a good theoretical basis and strong face validity. Some trials of recruiting opinion leaders to support the implementation of research findings have observed significant improvements in clinical care. However, this study has highlighted some of the likely problems of recruiting opinion leaders. First, opinion leaders appear to be monomorphic – separate identification exercises would be required for each clinical area or targeted behaviour. Second, the identification of opinion leaders and their coverage, if the underlying social networks were highly variable and idiosyncratic (except in the national specialty groups), suggests that recruitment of opinion leaders is unlikely to be an effective general strategy across all settings and professional groups. The more specialised the group, the more opinion leaders may be a useful strategy.

## Authors' contributions

Conception (JMG, TI, MPE, JK), Design (JMG, TI, MPE, JK, FS), Conduct (JMG, TI, MPE, JK, FS, JG, GM), Analysis (JMG, TI, JG, GM), Writing (MPE, JMG). All authors commented on successive drafts of the paper.
